# Increased functional connectivity between presupplementary motor area and inferior frontal gyrus associated with the ability of motor response inhibition in obsessive–compulsive disorder

**DOI:** 10.1002/hbm.25699

**Published:** 2021-11-24

**Authors:** Hirofumi Tomiyama, Keitaro Murayama, Kiyotaka Nemoto, Mayumi Tomita, Suguru Hasuzawa, Taro Mizobe, Kenta Kato, Aikana Ohno, Sae Tsuruta, Osamu Togao, Akio Hiwatashi, Tomohiro Nakao

**Affiliations:** ^1^ Department of Neuropsychiatry, Graduate School of Medical Sciences Kyushu University Fukuoka Japan; ^2^ Department of Neuropsychiatry, Faculty of Medicine University of Tsukuba Tsukuba Japan; ^3^ Department of Psychology Kurume University Kurume Japan; ^4^ Graduate School of Human‐Environment Studies Kyushu University Fukuoka Japan; ^5^ Department of Clinical Radiology, Graduate School of Medical Sciences Kyushu University Fukuoka Japan

**Keywords:** cingulo‐opercular salience network, cortico‐striato‐thalamo‐cortical circuit, fronto‐striatal circuit, inferior frontal gyrus, obsessive–compulsive disorder, presupplementary motor cortex, response inhibition, resting‐state functional MRI, stop‐signal task, ventral attention cortico‐striato‐thalamo‐cortical circuit

## Abstract

Recent evidence suggests that presupplementary motor area (pre‐SMA) and inferior frontal gyrus (IFG) play an important role in response inhibition. However, no study has investigated the relationship between these brain networks at resting‐state and response inhibition in obsessive–compulsive disorder (OCD). We performed resting‐state functional magnetic resonance imaging scans and then measured the response inhibition of 41 medication‐free OCD patients and 49 healthy control (HC) participants by using the stop‐signal task outside the scanner. We explored the differences between OCD and HC groups in the functional connectivity of pre‐SMA and IFG associated with the ability of motor response inhibition. OCD patients showed a longer stop‐signal reaction time (SSRT). Compared to HC, OCD patients exhibit different associations between the ability of motor response inhibition and the functional connectivity between pre‐SMA and IFG, inferior parietal lobule, dorsal anterior cingulate cortex, insula, and anterior prefrontal cortex. Additional analysis to investigate the functional connectivity difference from the seed ROIs to the whole brain voxels revealed that, compared to HC, OCD exhibited greater functional connectivity between pre‐SMA and IFG. Also, this functional connectivity was positively correlated with the SSRT score. These results provide additional insight into the characteristics of the resting‐state functional connectivity of the regions belonging to the cortico‐striato‐thalamo‐cortical circuit and the cingulo‐opercular salience network, underlying the impaired motor response inhibition of OCD. In particular, we emphasize the importance of altered functional connectivity between pre‐SMA and IFG for the pathophysiology of motor response inhibition in OCD.

## Graphical abstract

Obsessive–compulsive disorder patients had significantly different associations between the abilities of motor response inhibition and the resting‐state functional connectivity from pre‐SMA to IPL, IFG, dACC and anterior‐insula. Additionally, compared to healthy control, OCD exhibited greater functional connectivity between pre‐SMA and IFG, and this functional connectivity was correlated with the the abilities of motor response inhibition.

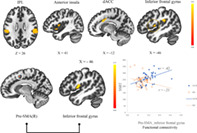

## 1 INTRODUCTION

Obsessive–compulsive disorder (OCD) is characterized by persistent intrusive thoughts and repetitive actions. A recent neurocognitive model of OCD considers that impairments in response inhibition and cognitive flexibility are central neurocognitive mechanisms of OCD and related disorders (Watanabe et al., 2015; Xu et al., 2016). In particular, OCD patients and their unaffected relatives both showed impaired response inhibition as measured by the stop‐signal task (SST) (Bora, 2020; Cavedini, Zorzi, Piccinni, Cavallini, & Bellodi, 2010; De Wit et al., 2012). Response inhibition could be an especially important trait to understand the biological basis of OCD. Response inhibition consists of three separable cognitive subcomponents including interference inhibition, action withholding, and motor response inhibition (action cancelation). SST is a representative paradigm to investigate the motor response inhibition (action cancelation), which asks the subject to inhibit an already started response by presenting a Stop‐stimulus immediately after a Go‐stimulus. The symptoms of OCD are characterized by failure to inhibit certain obsessive behaviors, and meta‐analyses have shown that OCD patients show impaired motor response inhibition (Snyder, Kaiser, Warren, & Heller, 2015). However, the biological basis of this dysfunction is not fully understood.

Neuroimaging studies have related OCD to alterations in parallel and partly segregated cortico‐striato‐thalamo‐cortical (CSTC) circuits and fronto‐parietal and fronto‐limbic networks (Stein et al., 2019). These circuits might be related to the compulsive symptoms and some cognitive dysfunctions of OCD (de Vries et al., 2017; Harrison et al., 2013; Pauls, Abramovitch, Rauch, & Geller, 2014; Thorsen et al., 2018; Thorsen et al., 2020; van den Heuvel et al., 2016). Especially, parallel and partly segregated CSTC circuits mediate some behavioral control functions involving motor, cognitive, affective, and motivational process (Stein et al., 2019; van den Heuvel et al., 2016; van Velzen, Vriend, de Wit, & van den Heuvel, 2014). In a recent meta‐analysis of task‐functional magnetic resonance imaging (fMRI) using broad inhibitory control paradigms (e.g., stop‐signal, go/no‐go, Stroop, Simon, flanker, anti‐saccade, and multisource interference task), OCD showed altered recruitment of the frontal and parietal regions, dorsal anterior cingulate cortex (dACC), and striatum (Norman et al., 2019) within these CSTC circuits and the fronto‐parietal network. In healthy individuals, the inhibition‐related prefrontal‐basal‐ganglia circuit including the right inferior frontal gyrus (IFG), presupplementary motor area (pre‐SMA), striatum, subthalamic nucleus, and globus pallidum are critical for motor response inhibition (action cancelation) (Aron, Robbins, & Poldrack, 2014; Chambers, Garavan, & Bellgrove, 2009). It is hypothesized that a neural stop‐signal is sent from IFG and pre‐SMA to the motor cortex through cortico‐striatal‐thalamic‐cortical projection to inhibit motor response (Chambers et al., 2009; Jahanshahi, Obeso, Rothwell, & Obeso, 2015). Especially, the right IFG and right pre‐SMA are key components in response inhibition (Aron, Fletcher, Bullmore, Sahakian, & Robbins, 2003; Chambers et al., 2006), and previous literature showed that high activation of IFG (Aron, Behrens, Smith, Frank, & Poldrack, 2007) and pre‐SMA (Chao, Luo, Chang, & Li, 2009) is related to good performance on the SST by healthy individuals. In OCD, the most consistent finding is decreased activation of IFG and striatum during SST, but the sample size is very limited (Kang et al., 2013; Rubia et al., 2010; Woolley et al., 2008). In the largest study to date, OCD patients and their unaffected relatives showed greater activation of pre‐SMA and reduced activation of IFG during SST, which could be a compensatory mechanism for neural inefficiency as a genetic vulnerability of OCD (De Wit et al., 2012). Though the same study group performed psychophysiological interaction analyses and dynamic causal modeling, they failed to show evidence for altered connectivity between pre‐SMA and IFG during SST (van Velzen et al., 2014). Although the pre‐SMA and IFG might play especially important roles during response inhibition, the characteristics of baseline resting‐state functional network underlying abnormal activations during SST in OCD are not fully understood.

In contrast to task‐fMRI, which is primarily focused on interpreting the activation of brain regions that are stimulated by cognitive task performance, resting‐state fMRI (rsfMRI) measures the temporal coupling between many brain areas during rest (Fox & Raichle, 2007). It is suggested that these resting‐state functional brain networks affect the characteristics of brain activation during cognitive tasks (Cole, Bassett, Power, Braver, & Petersen, 2014). These spontaneous blood‐oxygen level‐dependent (BOLD) signals may reflect actual neuronal activity in the resting state (Ma et al., 2016; Matsui, Murakami, & Ohki, 2016; Shmuel & Leopold, 2008). Measuring the relationships between resting‐state functional brain networks and cognitive function is useful for linking the intrinsic fluctuations in neural activity with cognitive function, addressing a more comprehensive understanding of human cognitive processing (Sheffield & Barch, 2016). There is only one previous study that investigated the relationship between the resting‐state functional network and the ability to inhibit motor response in OCD, and it reported that greater functional connectivity between the middle cingulate cortex and parahippocampal cortex is associated with the impaired motor response inhibition (Kang et al., 2013). However, these results may be affected by the very limited sample size (18 OCD patients) and methodological issues. The authors examined only the connectivity between brain regions that showed abnormal activation during SST, and they did not examine the functional connectivity of pre‐SMA or IFG. It is still unknown whether there is a characteristic resting‐state functional connectivity of pre‐SMA and IFG associated with the impaired motor response inhibition in OCD.

In this study, we focused on motor response inhibition (action cancelation). Deficits in motor response inhibition could represent candidate endophenotypes for OCD and be related to OCD symptoms. There was no previous study that investigated the association between the ability of motor response inhibition and the resting‐state functional connectivity of pre‐SMA and IFG. Investigation of the resting‐state functional network has the advantage of identifying core abnormalities that are not the result of effort or strategy, and will provide a reliable measure of baseline brain activity (Gusnard & Raichle, 2001). Considering its relatively low burden of data acquisition, resting‐state fMRI may be a promising tool for comprehensive understanding of the association between varying characteristics of brain functional network and the cognitive system in psychiatric illness (Insel et al., 2010). To investigate the association between the measures that come from independent sources (resting‐state fMRI and the motor response inhibition measured by SST outside the scanner) could provide novel insight regarding the underlying brain process for impaired response inhibition in OCD. We aimed to determine whether there are differences in resting‐state functional connectivity from pre‐SMA and IFG associated with motor response inhibition in OCD patients and healthy control (HC). We hypothesized that OCD patients would show altered functional connectivity from IFG and pre‐SMA to fronto‐striatal regions (IFG, pre‐SMA, striatum) within CSTC circuit responsible for response inhibition, and that these patterns of connectivity would be associated with impaired motor response inhibition in OCD.

## 2 METHODS

### 2.1 Subjects

We employed the same inclusion and exclusion criteria as described in our previous literature (Tomiyama et al., 2019). We recruited only OCD patients who were clinically diagnosed with no another Axis I disorder. OCD participants were interviewed by using the Structured Clinical Interview for DSM‐IV Axis I Disorders‐Patient Edition (SCID‐I/P) (First, Spitzer, Miriam, & Williams, 1995) and we ensured that all of them fulfilled DSM‐IV (American Psychiatric Association, 1994) criteria. We also confirmed that all patients also fulfilled DSM‐5 (American Psychiatric Association, 2013) criteria and that none of them met the criteria for any current comorbid Axis I disorder. All OCD patients had not taken any psychiatric medication for at least four weeks prior to the MRI scanning, and 15 OCD patients were drug‐naïve.

We recruited all HC participants from the local community and interviewed by using he Structured Clinical Interviews for DSM‐IV Non‐patient Edition (SCID‐I/NP) (First, Spitzer, Miriam, & Williams, 2002). We excluded HC participants who had a familial history of OCD because a recent endophenotype study suggested that first‐degree relatives of patients could have common brain dysfunctions associated with the ability to inhibit response (Chamberlain et al., 2007; De Wit et al., 2012). Also, we excluded the participants who had a lifetime history of significant head injury, seizure disorder, or intellectual disability. MRI scanning, clinical assessment and SST were conducted within a few hours on the same day. This study was approved by the Kyushu University Ethics Committee. Prior to the inclusion of this study, all participants provided written informed consent.

Consequentially, this study included 41 Japanese OCD patients (age 33.34 ± 11.74 (mean ± SD), 16 men and 25 women) and 49 HC participants (age 33.33 ± 10.36 (mean ± SD), 18 men and 31 women). There was no significant difference between OCD and HC groups in terms of age, gender and handedness.

### 2.2 Clinical assessment

We employed the same clinical assessment as described in our previous literature (Tomiyama et al., 2019). All OCD patients were interviewed by using the Japanese version of the Yale‐Brown Obsessive‐Compulsive Scale (Y‐BOCS) (Nakajima et al., 1995) to assess the severity of OCD symptoms. Partients with OCD have a moderate degree of severity in OCD symptoms with the scores of Y‐BOCS were 24.17 ± 6.25 (mean ± SD). Durations of illness were 12.33 ± 11.01 (mean ± SD) year. The Hamilton Ratings Scales for Anxiety (HAM‐A) (Hamilton, 1959) and Depression (17‐item HAM‐D) (Hamilton, 1960) were also used to examine the severity of anxiety and depression. We used the Japanese version of the National Adult Reading Test (JART) (Matsuoka, Uno, Kasai, Koyama, & Kim, 2006) to estimate a participant's intelligence quotient (IQ) (Table 1). Demographic and clinical data were statistically analyzed using χ^2^, Student's *t* test, and the Mann‐Whitney *U* test to detect group differences between OCD and HC.

**TABLE 1:** Demographic and clinical characteristics

			Statistics				
Variable	OCD (*n* = 41)	HC (*n* = 49)	*χ* ^2^	*t*	*u*	*df*	*p*‐Value
Demographic and clinical characteristics							
Sex, male/female	16/25	18/31	0.05			1	.831
Hand, right/left	36/5	47/2	2.05			1	.239
Age, years	33.34 (11.74)	33.33 (10.36)		0.01		88	.995
IQ^a^	103.50 (8.72)^a^ ^,^ ^b^	107.16 (9.92)		−1.82		87	.073
HAM‐D‐17	4.85 (4.83)	—					
HAM‐A	5.88 (7.67)	—					
Y‐BOCS total	24.17 (6.25)	—					
Y‐BOCS obsession	12.17 (3.50)	—					
Y‐BOCS compulsions	12.00 (3.60)	—					
Stanford sleepiness scale	3.4 (1.53)	3.12 (1.38)		0.92		91	.364
Stop‐signal task							
Successful inhibition (%)	48.68 (6.40)	50.38 (8.13)			880.5		.313
SSRT(ms)	262.70 (30.25)	239.83 (38.73)		2.52	638.5		.013*

*Note*: SSRT was expressed as median (interquartile deviation). Other variables were expressed as mean (*SD*, standard deviation), or n/n, as appropriate. *p < .05.

Abbreviations: HAM‐A, Hamilton Anxiety Scale; HAM‐D, Hamilton Depression Scale; IQ, intelligence quotient; JART, Japanese version of National Adult Reading Test; SSRT, stop‐signal reaction time; Y‐BOCS, Yale‐Brown Obsessive Compulsive Scale.

^a^ Estimated IQ was measured with the JART.

^b^ One participant did not complete JART.

### 2.3 Stop‐signal task

Response inhibition, which is the ability to stop an action according to the situation, was tested using the stop‐signal task (SST). After MRI scanning, participants performed SST outside the scanner. We used a computerized version of SST software (Verbruggen, Logan, & Stevens, 2008), which presents an experiment consisting of three blocks of 64 trials. Participants were instructed to press the right or left button to indicate the direction displayed on the computer screen as fast as possible. They were also instructed to stop their response when the stop‐signal sounded (750 Hz, 75 ms). This stop‐signal would appear at variable time intervals after a go signal. Twenty percent of all trials were stop‐signal trials. The stop‐signal delay (SSD) was initially set at 250 ms, adjusted continuously with the tracking procedure (if inhibition was successful, SSD was increased by 50 ms and if inhibition unsuccessful, SSD was decreased by 50 ms), and designed for a 50% successful inhibition outcome (Verbruggen & Logan, 2008). The stop‐signal reaction time (SSRT) is a measure of the speed of the inhibition. At the same time, a control score (percentage of successful inhibitions) to indicate that the task design functioned correctly was recorded. Additionally, for quality control of SST, we excluded the participants who made mistakes on more than 20% of go‐trials (Whelan et al., 2012) or who showed a stop‐trial error percentage under 25% or over 75% (Congdon et al., 2012).

### 2.4 Image data acquisition and preprocessing

We employed the same method for image data acquisition and preprocessing as described in our previous report (Tomiyama et al., 2019). MRI data was acquired by using a 3.0‐Tesla MRI scanner (Achieva TX, Phillips Healthcare, Best, The Netherlands) equipped with standard phased array head coils. Prior to MRI scanning, we instructed the participants to relax with their eyes open and watch a presented grey cross during the functional scanning. At first, we acquired T2*‐weighted gradient‐echo echo‐planar imaging (EPI) sequence (echo time (TE), 30 ms; repetition time (TR), 2500 ms; field of view (FOV), 212 × 212 mm; matrix, 64 × 64; slice thickness, 3.2 mm; flip angle, 80°). After an initial 10‐sec dummy scan, we acquired 240 functional volumes from a 10‐minute real scan. After EPI image scanning, we also acquired high‐resolution T1‐weighted anatomical images (TE = 3.8 ms; TR = 8.2 ms; FOV 240 × 240 mm; flip angle 8°; slice thickness, 1 mm; inversion time = 1026 ms). After acquisition of all MRI image data, all participants were interviewed by using the Stanford‐Sleepiness Scale to check the arousal level during functional image scanning. No participant fell asleep during the MRI scanning, and there was no significant difference between OCD and HC groups in arousal level during the functional scan (Table 1). For quality control, we manually checked all EPI and T1 images, and excluded the two participants (one OCD and one HC) whose EPI image of frontal areas had broad deficit due to artifacts.

Standard preprocessing and denoising pipelines of CONN toolbox (Whitfield‐Gabrieli & Alfronso, 2012) 17.f running on MATLAB R2016b (MathWorks, Inc., Natick, MA, USA) on MacOS 10.12.6 was conducted as previously described (Tomiyama et al., 2019). We discarded the first four volumes, and then, the remaining 236 functional volumes were preprocessed using standard preprocessing pipelines of CONN toolbox. Functional images were corrected for slice timing based on the slice order, and realigned and normalized in accordance with the standard Montreal Neurological Institute (MNI) template. We estimated the six rigid‐body parameters (translational and rotational) for each participant. To exclude image artifacts due to head movement, we used the ART scrubbing procedure (https://www.nitrc.org/projects/artifact_detect/) using the 97th percentile in a normative sample (with thresholds for motion = 0.9 mm and global signal *z* = 5). Signal noises from the white matter, cerebrospinal fluid, and the parameters of head movement were denoised. Then, functional images were band‐pass filtered at 0.008–0.09 Hz, and smoothed with a Gaussian kernel of 6‐mm full width at half‐maximum. There was no significant difference between OCD and HC groups in motion parameter (mean motion [*t*(88) = −.559 *p* = .680]), mean global blood‐oxygen‐level‐dependent (BOLD) signal [*t*(88) = .094, *p* = .914]) and invalid scans due to scrubbing procedure (*t* = .92, *p* = .361).

### 2.5 Data analysis

Following the preprocessing steps, the BOLD signal time series correlation was calculated between each pair of sources for each participant across the resting‐state time series, and then a Fisher *z* transformation was applied. Seed‐based connectivity maps were generated from each seed region of interest (ROI) for each participant. We created the seed ROIs as a 4 mm radius sphere (diameter = 8 mm) at MNI coordinates at right pre‐SMA (+10,10,50) (Xu et al., 2016) and right IFG (+61,21,13) (Chambers et al., 2006) based on previous brain stimulation studies which estimated a direct relationship between brain regions and the cognitive function. We did not find the coordinates of left pre‐SMA and left‐IFG in previous literature using brain stimulation; therefore, we created the seed ROIs of left pre‐SMA and IFG based on the task‐functional MRI study using SST (De Wit et al., 2012) (left pre‐SMA [−15,14,67] and left IFG [−33,23,−8] as 4 mm radius sphere [diameter = 8 mm]).

We generated seed‐based connectivity maps from each seed ROI within each group and ensured that both groups showed the typical characteristic functional connectivity patterns that have been associated with the applied seed regions (Supplement Figure S1).

We examined group differences by voxel‐wise whole brain linear regression analysis associated with SSRT scores using an analysis of covariance interaction model, while controlling for age, gender, and IQ. (These variables are commonly controlled for when examining the relationship between brain and cognitive function (Posner et al., 2017; Vaghi et al., 2017).) Statistical significance was set at a voxel height threshold of *p* < .001, and cluster‐level threshold of *p* < .05 false discovery rate (FDR) corrected (nonparametric statistics (simulation 5,000) based on the permutation/randomization analyses (Bullmore et al., 1999), supplied with the CONN toolbox (Alfronso, 2020)). Beta values showing significant difference clusters were examined to determine the strength and direction of correlation with the SSRT scores within each group.

For better interpretation of the main results, we examined the group differences between OCD and HC in functional connectivity between the seed ROIs and the whole brain voxels, while controlling for age, gender, and IQ. Statistical significance was set at a voxel height threshold of *p* < .001, and a cluster‐size threshold of *p* < .05 FDR corrected. Then, we conducted correlation analyses between the functional connectivity value showing significant difference clusters from each ROI and the SSRT scores within each group.

## 3 RESULTS

### 3.1 Between‐group differences in SST

There was no difference between OCD and HC groups in the control score (percentage of successful inhibitions) in SST (Table 1). There was no participant who mistook more than 20% in go‐trials (OCD: 0.74 ± 2.59 [mean ± *SD*], HC: 0.04 ± 0.17 [mean ± *SD*]) (Whelan et al., 2012) or who showed stop‐trial error percentage under 25% or over 75% (OCD: 51.32 ± 6.32 [mean ± *SD*]), HC: 49.62 ± 8.13 [mean ± *SD*]) (Congdon et al., 2012); therefore, no participant was excluded due to SST results. OCD patients showed longer SSRT compared with the HC (*p* < .05) (OCD: 262.700 ± 30.25 [mean ± *SD*]), HC: 239.83 ± 21.38 [mean ± *SD*]) (Table 1). To check the effects of psychiatric symptom severity on the SST performance of the OCD group, we analyzed the correlation between SSRT and Y‐BOCS total scores, HAM‐D scores, and HAM‐A scores. There was no significant correlation between SSRT and these clinical scores.

### 3.2 Different relationships between functional connectivity and SSRT in OCD and HC

**FIGURE 1:**
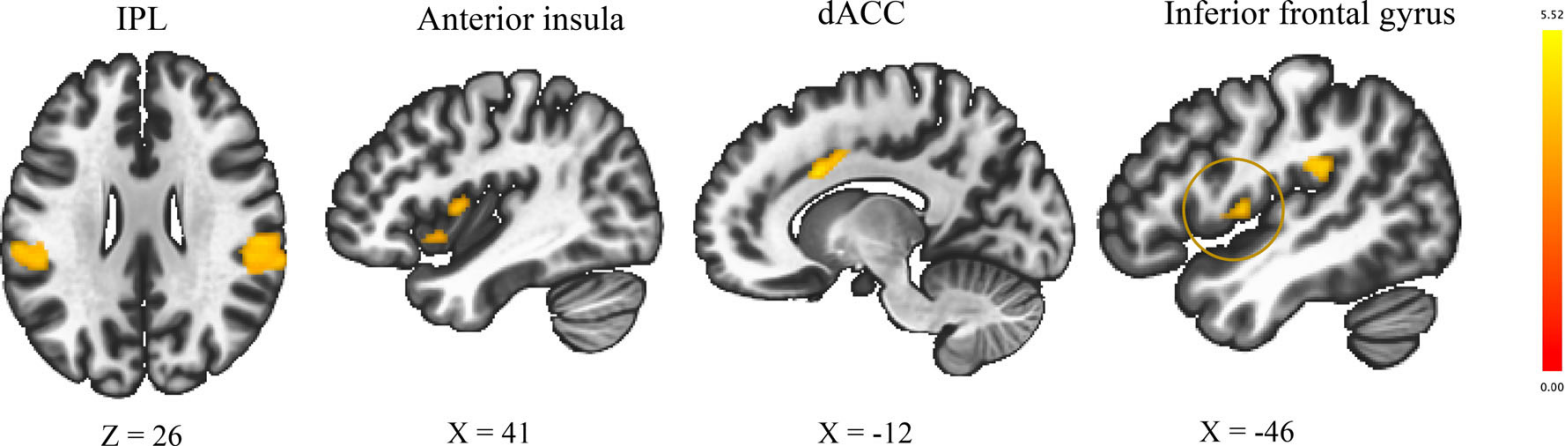
Brain areas, including inferior frontal gyrus, in which increased functional connectivity with the right presupplementary‐motor area (pre‐SMA) was found to be significantly related to worse motor response inhibition in obsessive–compulsive disorder (OCD) compared to healthy control (HC) (cluster‐level corrected significance *p* < .05 false discovery rate (FDR), after applying a per‐voxel height threshold of *p* < .001, nonparametric status [permutation/randomization analysis]). dACC, dorsal anterior cingulate cortex; IPL, inferior parietal lobule

A significant group difference was found in the association between SSRT scores and functional connectivities between the right pre‐SMA and bilateral inferior parietal lobule (IPL; cluster peaking at parietal operculum cortex extending to supramarginal gyrus), bilateral IFG (cluster peaking at central operculum cortex), dACC, anterior‐insula, and right anterior prefrontal cortex (Table 2, Figure 1). In the OCD group, greater functional connectivities between these brain areas were associated with higher SSRT scores (functional connectivity between right pre‐SMA and bilateral IPL [*r* = .57 and .60], bilateral IFG [*r* = .59 and .56], dACC [*r* = .65], bilateral insula [*r* = .60 and .59], and right anterior prefrontal cortex [*r* = .56]). In the HC group, there was no correlation between these functional connectivities and the SSRT scores (functional connectivity between right pre‐SMA and bilateral IPL [*r* = −.34 and −.31], bilateral IFG [*r* = −.16 and −.27], dACC [*r* = −.21], bilateral insula [*r* = −.18 and −.22], and right anterior prefrontal cortex [*r* = −.27]). There was no significant difference between OCD and HC in functional connectivity from left pre‐SMA and bilateral IFG ROIs associated with SSRT scores.

**TABLE 2:** Different relationships between functional connectivity and SSRT in OCD and HC

Seed	Region	*Ke*	*x*	*y*	*z*	Direction	*p*‐FDR^a^	Effect size
L. pre‐SMA	—	—	—	—	—	—	—	—
R. pre‐SMA	R. insula cortex	301	32	12	6	OCD > HC	.00067*	.016
	L. inferior frontal gyrus (opercular part), insula cortex	207	−42	−2	12	OCD > HC	.0014*	.047
	R. inferior frontal gyrus (opercular part)	145	50	0	0	OCD > HC	.0036*	.014
	R. inferior parietal lobule	898	66	−30	22	OCD > HC	.00000*	.009
	L. inferior parietal lobule	268	−58	−28	22	OCD > HC	.00079*	.003
	R. anterior prefrontal cortex	200	32	42	10	OCD > HC	.0014*	.000
	L. dorsal anterior cingulate cortex	165	−14	12	30	OCD > HC	.0027*	.010
	R. dorsal anterior cingulate cortex	151	8	12	38	OCD > HC	.0032*	.007
	L. insula cortex	68	−36	−4	−8	OCD > HC	.035	.006
L. IFG	—	—	—	—	—	—		—
R. IFG	—	—	—	—	—	—		—

*Note*: *p‐FDR < .0125 (representing a Bonferroni‐corrected *p*‐value adjusted for four comparisons after cluster‐level FDR correction). Peak coordinates are given in MNI space.

Abbreviations: BA, Broadmann area; FDR, false discovery rate; HC, healthy control; IFG, inferior frontal gyrus; *Ke*, cluster extent; L, left; MNI, Montreal Neurological Institute; OCD, obsessive–compulsive disorder; R, right; pre‐SMA, presupplementary motor area; SSRT, stop‐signal reaction time.

^a^ Cluster‐level corrected *p* < .05 FDR after applying a voxel height threshold of *p* < .001.

### 3.3 Functional connectivity differences from seed ROIs to whole brain voxels between OCD and HC

**FIGURE 2:**
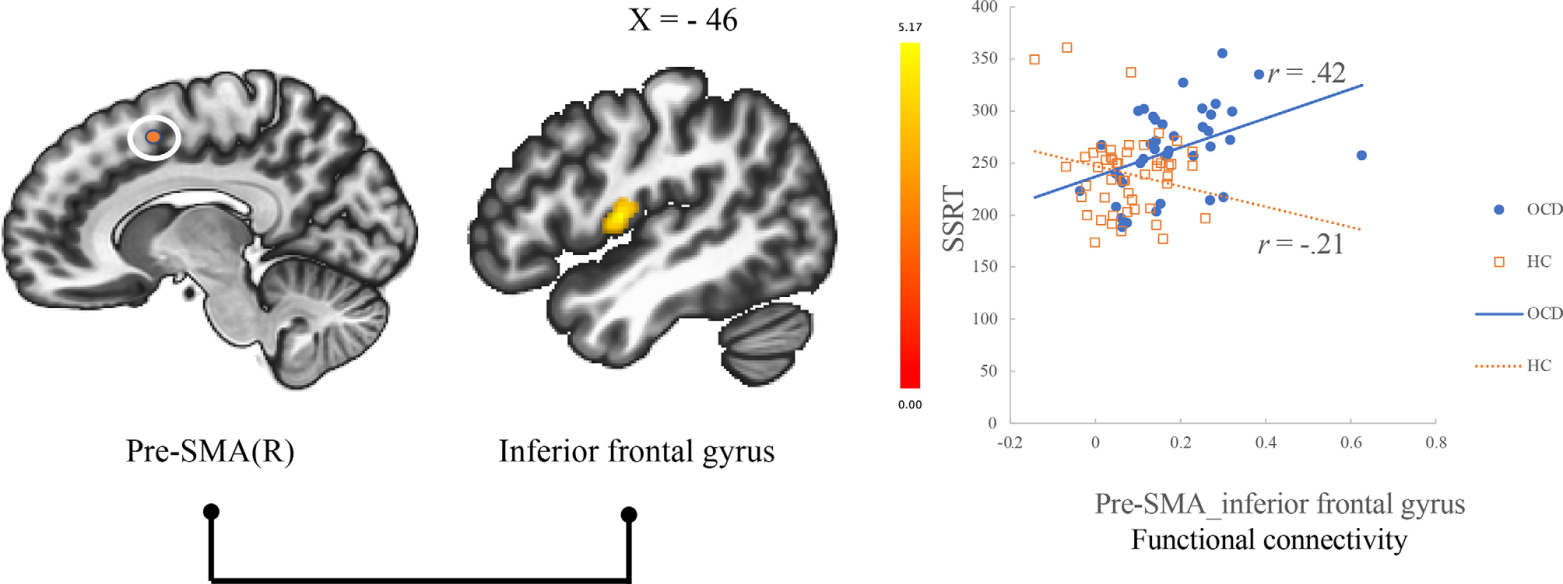
Greater functional connectivity between pre‐SMA and IFG was associated with more impaired response inhibition in the obsessive–compulsive disorder (OCD) group. *r* = Spearman's rank‐order correlation coefficient. IFG, inferior frontal gyrus; L, left; pre‐SMA, presupplementary‐motor area; R, right; SSRT; stop‐signal reaction time

Between‐group connectivity comparisons revealed that, compared with HCs, OCD patients showed significantly greater functional connectivity between right pre‐SMA and IFG (cluster peaking at central operculum cortex) (Table 3). Correlation analysis revealed that, within the OCD group, functional connectivity between these areas was positively correlated with longer SSRT (*r* = .42) (Figure 2). There was no significant difference between OCD and HC in functional connectivity from left pre‐SMA and bilateral IFG ROIs.

**TABLE 3:** Functional connectivity differences between OCD and HC

Seed	Region	*Ke*	*x*	*y*	*z*	Direction	*p*‐FDR^a^	Effect size
L. pre‐SMA	—	—	—	—	—	—		—
R. pre‐SMA	L. inferior frontal gyrus	252	−46	−4	10	OCD > HC	.00079*	.179
L. IFG	—	—	—	—	—	—		—
R. IFG	—	—	—	—	—	—		—

*Note*: *p‐FDR < .0125 (representing a Bonferroni‐corrected *p*‐value adjusted for four comparisons after cluster‐level FDR correction). Peak coordinates are given in MNI space.

Abbreviations: BA, Broadmann area; FDR, false discovery rate; HC, healthy control; IFG, inferior frontal gyrus; *Ke*, cluster extent; L, left; MNI, Montreal Neurological Institute; OCD, obsessive–compulsive disorder; R, right; pre‐SMA, presupplementary motor area.

^a^ Cluster size corrected *p* < .05 FDR after applying a voxel height threshold of *p* < .001.

## 4 DISCUSSION

Compared with HC, OCD patients showed worse motor response inhibition, in line with the previous finding (Menzies et al., 2007; Snyder et al., 2015). OCD patients had significantly different associations between the functional connectivity from pre‐SMA to IPL, IFG, dACC, anterior‐insula, and anterior prefrontal cortex, and the abilities of motor response inhibition (Table 2, Figure 1). Additionally, in between‐group connectivity comparisons between the seed ROIs and the whole brain voxels, OCD patients showed greater functional connectivity between pre‐SMA and IFG (Table 3), and this altered connectivity was correlated with more impaired response inhibition (Figure 2). These results suggested that impaired motor response inhibition of OCD is related to the resting‐state functional connectivity between pre‐SMA and IFG and the regions of cingulo‐opercular salience network (SN). Our results especially emphasize the importance of altered functional connectivity between pre‐SMA and IFG for the motor response inhibition in OCD.

The primary findings of this study were that OCD patients exhibited a different association between the SSRT scores and the functional connectivity between pre‐SMA and some brain areas including IFG (Table 2, Figure 1) compared to HC. Further, compared to HC, OCD patients showed greater functional connectivity between the right pre‐SMA and IFG, and this connectivity was correlated with more impaired response inhibition (Table 3, Figure 2). Both results indicated that the functional connectivity between pre‐SMA and IFG is related to impaired motor response inhibition in OCD patients. Many literatures showed that pre‐SMA and IFG are essential regions of CSTC circuit responsible for motor response inhibition (Chambers et al., 2009). It has been shown that there are functional and structural connections between pre‐SMA and IFG for stopping an action (Swann et al., 2012). It is hypothesized that a neural stop‐signal is sent from IFG and pre‐SMA to the motor cortex through the cortico‐striatal‐thalamic‐cortical projection to achieve motor response inhibition (Aron, 2011; Chambers et al., 2009; Duann, Ide, Luo, & Li, 2009). Alterations of IFG (De Wit et al., 2014) and pre‐SMA (D'Urso et al., 2016) are thought to be implicated in OCD pathophysiology. Previous literature showing that deficits in motor response inhibition (action cancelation) were associated with gray matter volume in the IFG and SMA (Menzies et al., 2007) could also support our results. In a task fMRI study using SST, OCD patients showed hyperactivation in pre‐SMA and showed decreased activation in IFG, which could reflect neural inefficiency of these brain areas (De Wit et al., 2012). Meta‐analysis also showed that OCD exhibited greater activation of SMA and reduced activation of IFG (frontal operculum area) during an inhibitory control task including SST (Norman et al., 2019). Our results of greater resting‐state functional connectivity between pre‐SMA and IFG may be an underlying mechanism for this abnormal activation during response inhibition. Brain stimulation is promising as an add‐on treatment of refractory OCD (Rapinesi et al., 2019). Recent literature showed that rTMS to the pre‐SMA (Obeso et al., 2017) could improve the response inhibition in healthy individuals, and transcranial direct current stimulation to pre‐SMA could improve OCD symptoms (D'Urso et al., 2016). Greater resting‐state functional connectivity between pre‐SMA and IFG could be an important target for developing a new treatment for the impaired motor response inhibition of OCD.

We found that, compared to HC, OCD exhibited different associations between the SSRT scores and the functional connectivity between pre‐SMA and IPL, anterior‐insula, dACC, and anterior prefrontal cortex (Table 2, Figure 1). These brain regions are overlapped with those observed in task‐fMRI studies of broad types of inhibitory control (Zhang, Geng, & Lee, 2017). Pre‐SMA, dACC, anterior‐insula (Uddin, 2015), and the anterior part of IPL (Igelstrom & Graziano, 2017) are central nodes of the cingulo‐opercular SN (Peters, Dunlop, & Downar, 2016), and nearly corresponds to the more anterior subnetwork of the ventral attention network. It is hypothesized that SN assists other brain regions in generating appropriate behavioral responses, and facilitates rapid access to the motor network (Menon & Uddin, 2010), and that error signal from the SN contributes to behavioral correction during errors on broad inhibitory control tasks (Norman et al., 2019; Zhang et al., 2017). A recent meta‐analysis of task‐fMRI using broad inhibitory control task (e.g., stop‐signal, go/no‐go, Stroop, Simon, flanker, anti‐saccade, and multisource interference task) showed that, in patients with OCD, error signal from SN does not efficiently increase activation of CSTC circuit responsible for inhibitory control, which lead hyperactivation of SN and hypoactivation of CSTC circuit (Norman et al., 2019). Also, a recent meta‐analysis of rsfMRI showed that OCD have alterations of resting‐state large‐scale intrinsic networks including SN (Gursel, Avram, Sorg, Brandl, & Koch, 2018), and this alteration might be associated with task‐related disrupted activation during broad inhibitory control process of OCD. Our results may suggest that characteristics of resting‐state functional network between pre‐SMA and some cortical regions within SN in OCD are underlying mechanisms of abnormal activations during the motor response inhibition task.

In our HC group, there was no correlation between the SSRT scores and the functional connectivity between the right pre‐SMA and cortical areas including bilateral IPL, IFG, anterior‐insula, dACC, and right anterior prefrontal cortex. These brain areas are known to be activated during motor response inhibition (action cancelation) tasks as shown in meta‐analysis of task‐fMRI in healthy individuals (Zhang et al., 2017). At first glance, our results of HC seem inconsistent with that task‐fMRI literature; however, our results may suggest that resting‐state functional connectivity between cortical regions alone does not explain the ability of motor response inhibition. There is a possibility that functional connectivity of other subcortical areas, not between cortical areas, may be specifically associated with the ability of motor response inhibition of healthy individuals. Previous literature, showing that modulating for increased overall resting‐state functional connectivity of fronto‐basal‐ganglia circuit (functional connectivity between pre‐SMA and IFG and between IFG and striatum, subthalamic nucleus and globus pallidum) leads to shorter SSRT in healthy individuals (Xu et al., 2016), emphasized the importance of resting‐state functional connectivity between cortical regions and basal‐ganglia, not only between cortical regions. However, our method did not aim primarily to investigate the functional network associated with the ability of motor response inhibition in the HC group, and we could not conclude this issue from this study. Additionally, it is suggested that task‐evoked activity is not independent from resting‐state spontaneous functional connectivity (Hoffstaedter et al., 2014; Smith et al., 2009); however, the relationship between them is not straightforward (He, 2013; Lynch et al., 2018). To date, there is a lack of consensus about the relationship between task‐evoked activation and resting‐state baseline activity. It is not fully understood which resting‐state functional network is particularly associated with motor response inhibition in healthy individuals. Future study to investigate the resting‐state functional network associated with better response inhibition in healthy individuals is needed.

There are several limitations in this study. First, we examined only SST among many cognitive domains; therefore, we could not determine whether our findings are specifically associated with the impaired response inhibition or associated with broad cognitive impairments of OCD. Second, the sample size was relatively small. Future work that includes a larger sample size is needed. Third, we did not consider OCD symptom heterogeneity. There is a possibility that different OCD symptom dimensions could be caused by different cognitive deficits and biological traits (Harrison et al., 2013; Hashimoto et al., 2011; Reess et al., 2018). However, the previous literature showed that impaired response inhibition is independent of the symptom dimension of OCD (Lei et al., 2015). Thus, our findings could show the essential neural bases associated with impaired response inhibition regardless of the symptom dimensions of OCD. Fourth, we investigated the indirect relationship between the resting‐state functional network and the ability of motor response inhibition instead of investigating a direct relationship using the task‐fMRI method; therefore, it is slightly difficult to speculate about the meaning of the results in HC groups. Future work is needed to explore the relationship between task‐evoked activity and the spontaneous resting‐state functional connectivity associated with motor response inhibition, using both task‐fMRI and resting‐state fMRI in the same subjects. Fifth, our OCD patients had a relatively long duration of illness, and we could not exclude a possibility that they are potentially suffered any personality disorder. Comorbidity of personality disorder may be related to poor prognosis (Starcevic & Brakoulias, 2017) and lead to long duration of illness. Also, six OCD patients had past treatment history of behavioral therapy, and we could not completely exclude the effect of these past psychological treatment. Finally, we recruited only OCD patients who have no another Axis I disorder to investigate core neural underpinning associated with the OCD itself. However, it is common that OCD patients have another mood or anxiety disorder (Ruscio, Stein, Chiu, & Kessler, 2010). From these limitations, we could not generalize our results. Our results need to be replicated in the future study, and future work is needed to identify the effect of the long duration of illness, comorbid personality traits, and comorbidity of other mood and anxiety disorders.

In conclusion, our results showed that, compared to HC, OCD exhibits different associations between the ability of motor response inhibition and the functional connectivity of the regions of the CSTC circuit and the cingulo‐opercular SN. These results could provide additional insight into the intrinsic fluctuations in neural activity underlying the impaired motor response inhibition of OCD. Additionally, our results especially emphasize the importance of the resting‐state functional connectivity between pre‐SMA and IFG in the pathophysiology of impaired motor response inhibition (action cancelation) in OCD.

## CONFLICT OF INTEREST

The authors declare no potential conflict of interests.

## Supporting information


**Appendix S1:** Supplementary Information

## Data Availability

The data that support the findings of this study are available on request from the corresponding author upon reasonable request. The data are not publicly available due to ethical restrictions.
