# Supplementary material for: Increased functional connectivity between presupplementary motor area and inferior frontal gyrus associated with the ability of motor response inhibition in obsessive–compulsive disorder
Source: Hum Brain Mapp. 2021 Nov 24;43(3):974–84. doi: 10.1002/hbm.25699 (PMC8764470; doi:10.1002/hbm.25699)
Supplement: Supplementary file 1 — Appendix S1: Supplementary Information [file HBM-43-974-s001.docx]

Supplementary Figure S1: Within group effect from each seed ROIs
